# Research priorities to inform “Treat All” policy implementation for people living with HIV in sub‐Saharan Africa: a consensus statement from the International epidemiology Databases to Evaluate AIDS (IeDEA)

**DOI:** 10.1002/jia2.25218

**Published:** 2019-01-18

**Authors:** Marcel Yotebieng, Ellen Brazier, Diane Addison, April D Kimmel, Morna Cornell, Olivia Keiser, Angela M Parcesepe, Amobi Onovo, Kathryn E Lancaster, Barbara Castelnuovo, Pamela M Murnane, Craig R Cohen, Rachel C Vreeman, Mary‐Ann Davies, Stephany N Duda, Constantin T Yiannoutsos, Rose S Bono, Robert Agler, Charlotte Bernard, Jennifer L Syvertsen, Jean d'Amour Sinayobye, Radhika Wikramanayake, Annette H Sohn, Per M von Groote, Gilles Wandeler, Valeriane Leroy, Carolyn F Williams, Kara Wools‐Kaloustian, Denis Nash, the IeDEA Treat All in sub‐Saharan Africa Consensus Statement Working Group, Keri Althoff, Craig R. Cohen, Geraldina Dominguez, Stephany N. Duda, Aimee Freeman, Antoine Jaquet, April D. Kimmel, Kathryn E. Lancaster, Janne Markus, Rosemary McKaig, Pamela M. Murnane, Dominique Nsonde, Angela M. Parcesepe, Per M. von Groote, Rachel C. Vreeman, Carolyn F. Williams, Constantin Yiannoutsos

**Affiliations:** ^1^ The Ohio State University Columbus OH USA; ^2^ Institute for Implementation Science in Population Health City University of New York New York NY USA; ^3^ Department of Epidemiology and Biostatistics Graduate School of Public Health and Health Policy City University of New York New York NY USA; ^4^ Department of Health Behavior and Policy Virginia Commonwealth University School of Medicine Richmond VA USA; ^5^ Centre for Infectious Disease Epidemiology& Research School of Public Health & Family Medicine University of Cape Town Cape Town South Africa; ^6^ Institute of Global Health University of Geneva Geneva Switzerland; ^7^ University of North Carolina at Chapel Hill Chapel Hill NC USA; ^8^ Infectious Diseases Institute Makerere University Kampala Uganda; ^9^ Center for AIDS Prevention Studies Department of Medicine University of California San Francisco San Francisco CA USA; ^10^ Department of Obstetrics, Gynecology & Reproductive Sciences Bixby Center for Global Reproductive Health University of California San Francisco San Francisco CA USA; ^11^ Department of Pediatrics Indiana University School of Medicine Indianapolis IN USA; ^12^ School of Public Health and Family Medicine Faculty of Health Sciences University of Cape Town Cape Town South Africa; ^13^ Vanderbilt University School of Medicine Nashville TN USA; ^14^ Fairbanks School of Public Health Indianapolis IN USA; ^15^ Inserm Centre INSERM U1219‐Epidémiologie‐Biostatistique School of Public Health (ISPED) University of Bordeaux Bordeaux France; ^16^ Department of Anthropology University of California at Riverside Riverside CA USA; ^17^ Rwanda Military Hospital Kigali Rwanda; ^18^ TREAT Asia amfAR – The Foundation for AIDS Research Bangkok Thailand; ^19^ Institute of Social and Preventive Medicine (ISPM) University of Bern Bern Switzerland; ^20^ Inserm (French Institute of Health and Medical Research) UMR 1027 Université Toulouse 3 Toulouse France; ^21^ Epidemiology Branch Division of AIDS at National Institute of Allergy and Infectious Diseases (NIAID) National Institute of Health (NIH) Rockville MD USA; ^22^ Indiana University School of Medicine Indianapolis IN USA

**Keywords:** Treat All, universal HIV treatment, 90‐90‐90 targets, sub‐Saharan Africa, implementation science

## Abstract

**Introduction:**

“Treat All” – the treatment of all people with HIV, irrespective of disease stage or CD4 cell count – represents a paradigm shift in HIV care that has the potential to end AIDS as a public health threat. With accelerating implementation of Treat All in sub‐Saharan Africa (SSA), there is a need for a focused agenda and research to identify and inform strategies for promoting timely uptake of HIV treatment, retention in care, and sustained viral suppression and addressing bottlenecks impeding implementation.

**Methods:**

The Delphi approach was used to develop consensus around research priorities for Treat All implementation in SSA. Through an iterative process (June 2017 to March 2018), a set of research priorities was collectively formulated and refined by a technical working group and shared for review, deliberation and prioritization by more than 200 researchers, implementation experts, policy/decision‐makers, and HIV community representatives in East, Central, Southern and West Africa.

**Results and discussion:**

The process resulted in a list of nine research priorities for generating evidence to guide Treat All policies, implementation strategies and monitoring efforts. These priorities highlight the need for increased focus on adolescents, men, and those with mental health and substance use disorders – groups that remain underserved in SSA and for whom more effective testing, linkage and care strategies need to be identified. The priorities also reflect consensus on the need to: (1) generate accurate national and sub‐national estimates of the size of key populations and describe those who remain underserved along the HIV‐care continuum; (2) characterize the timeliness of HIV care and short‐ and long‐term HIV care continuum outcomes, as well as factors influencing timely achievement of these outcomes; (3) estimate the incidence and prevalence of HIV‐drug resistance and regimen switching; and (4) identify cost‐effective and affordable service delivery models and strategies to optimize uptake and minimize gaps, disparities, and losses along the HIV‐care continuum, particularly among underserved populations.

**Conclusions:**

Reflecting consensus among a broad group of experts, researchers, policy‐ and decision‐makers, PLWH, and other stakeholders, the resulting research priorities highlight important evidence gaps that are relevant for ministries of health, funders, normative bodies and research networks.

## 1 Introduction

The World Health Organization's (WHO) “Treat All” guidance of September 2015, which recommended that all individuals be treated as soon as possible after HIV infection and diagnosis 1, was a true paradigm shift in HIV care and treatment 2. Preventing illness and death among people living with HIV and averting new infections by reducing onward HIV transmission, Treat All is recognized as the primary strategy for achieving the Joint United Nations Programme on HIV/AIDS (UNAIDS) 90‐90‐90 targets 3, 4, 5 (Box 1). In view of its potential contribution to ending AIDS as a public health threat, Treat All is being adopted in countries around the globe.

Box 1 **Key Terms and Definitions**

1 *Treat All*: The Treat All approach recognizes that HIV infection should be treated as soon as possible after diagnosis because all patients, regardless of their stage of infection, benefit clinically from early treatment of HIV. In addition, because reduction in HIV viral load to undetectable levels eliminates the risk of onward transmission, the Treat All approach has the potential to provide the population health benefit of reducing HIV incidence. Treat All therefore is inclusive of efforts to diagnose and treat all persons with HIV as soon as possible after HIV infection.

*UNAIDS 90‐90‐90 Targets*: In 2014, the Joint United Nations Programme on HIV/AIDS and partners set targets to diagnose 90% of the people living with HIV (PLWH), provide treatment to 90% of those diagnosed with HIV, and achieve viral suppression among 90% of those on treatment by 2020 to help end the AIDS epidemic by 2030.

*Key and Underserved Populations*: We use the term Key Populations to refer to groups of people who are more likely to be exposed to HIV or to transmit it, and whose engagement is critical to a successful HIV response 65. Depending on the epidemic context and setting, key populations may include infants/children, adolescents, younger adults, men, women, older adults, female sex workers (FSW), men who have sex with men (MSM), people who inject drugs (PWID), transgender (TG) individuals and migrant/mobile populations. “Underserved” refers to an unmet need for HIV care services, including both testing and treatment services.

**Figure 1:**
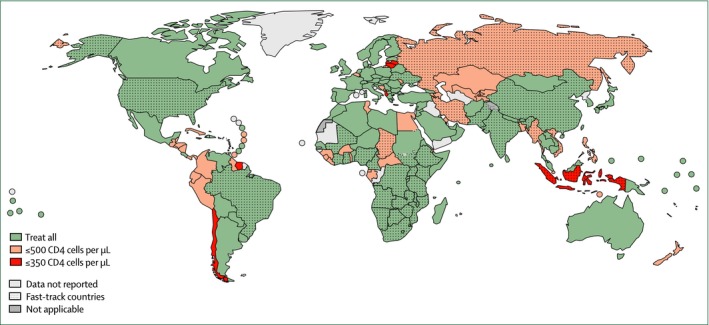
Uptake of national ‘Treat all’ policies for adults and adolescents with HIV, July 2017. (Source: WHO)

By 2017, most countries in sub‐Saharan Africa (SSA) had adopted some form of the Treat All recommendation (Figure 1) 2, 6, 7, 8. Nonetheless, new HIV infections and AIDS‐related mortality remain higher in SSA than other world regions, and the majority of people living with undiagnosed and untreated HIV infection live in SSA 9. To address high unmet need for HIV care and treatment and accelerate progress towards the UNAIDS 90‐90‐90 targets in SSA, policy‐makers and planners need evidence on effective strategies for promoting uptake of services and maximizing their impact on individual and population health outcomes, and on how best to address major bottlenecks impeding effective and efficient implementation of Treat All. To catalyze efforts to address these important evidence gaps, a global group of expert clinicians, researchers and programme specialists engaged in HIV research and service delivery in SSA undertook a multi‐step process to identify a set of initial research priorities with the potential to inform and guide Treat All implementation. With the rollout of Treat All accelerating in SSA in 2017, the aim of this process was to ensure that evidence on the implementation and scale‐up of Treat All policies is systematically gathered and examined to identify optimal programmatic strategies and to realize the individual and public health benefits of earlier HIV treatment more rapidly 10, 11, 12.

## 2 Methods

The Delphi method was used to develop and refine Treat All research priorities. The Delphi method is a flexible approach, widely used to reach consensus among experts in health research and other disciplines 13, 14, 15, 16, 17, 18. The Delphi method generally involves an iterative process of eliciting and aggregating opinions from a group of experts, with opportunities for participants to provide input during each round and to reassess and incorporate new insights and perspectives during subsequent rounds. A key feature of the Delphi method is that participants provide input independently during each round, resulting in a process that is not unduly influenced by any one individual or subset of participants 13, 14.

**Figure 2:**
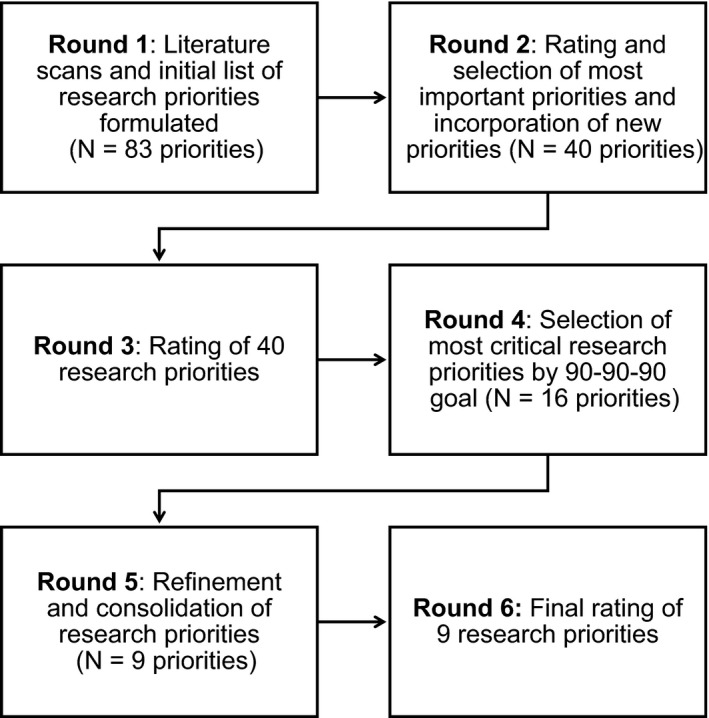
Consensus development process.

The process for reaching consensus around Treat All research priorities involved six phases, carried out between June 2017 and March 2018 (Figure 2). This process was led by a group of researchers involved in the International epidemiology Databases to Evaluate AIDS (IeDEA) consortium, a global collaboration that consolidates, curates and analyses longitudinal data on care and treatment of PLWH (Box 2).

Box 2 **IeDEA**

1 IeDEA is an international research consortium established in 2005 by the U.S. National Institute of Allergy and Infectious Diseases (NIAID) to provide a rich resource for globally diverse HIV/data (see www.iedea.org). The IeDEA Cohort Consortium collaborates to collect and harmonize clinical care data as a cost‐effective means of generating large data sets to address high priority research questions and streamline HIV/AIDS research.The Consortium includes 140 clinics (denoted by ⊙) in sub‐Saharan Africa that have provided care to more than 1 million PLWH. Representing 23 SSA countries, IeDEA is poised to provide some of the first data on the uptake and outcomes of Treat All implementation.
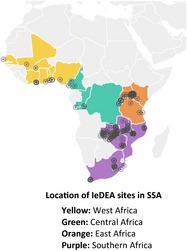

### 2.1 Round 1

A core group of 25 IeDEA investigators were invited to participate in a Treat All Consensus Statement Working Group, based on their expertise leading HIV research in 10 population‐specific and/or cross‐cutting content areas (see Box 3). Subsequently, 15 working group members reviewed and summarized the literature 2 in their respective areas of expertise and proposed an initial list of 83 research priorities across the 10 content areas.

Box 3 **Population‐specific and cross‐cutting content areas of expertise**

**Populations**

- Infants and children
- Adolescents
- Adults
- Pregnant and postpartum women
- Key populations (sex workers, people who inject drugs, men who have sex with men, mobile populations)

**Cross‐cutting content areas**

- Metrics and monitoring
- Models of care/strategies
- Policy modelling
- Mental health
- Substance use

### 2.2 Round 2

Via an online Qualtrics 19 survey, members of the working group were asked to anonymously rate (without ranking) the overall importance of each of the 83 proposed research priorities, using a scale of 1 (least important) to 5 (most important). Respondents were also asked to provide feedback on whether each research priority was sufficiently specific and clear, and to suggest additional research priorities in response to perceived gaps. Sixteen out of 25 working group members (64%) participated in the survey, and 32 out of 83 research priorities were rated both “high” in importance (i.e. a “4” or “5” rating) and clear/specific by at least two‐thirds of respondents. In addition, eight new research priorities were proposed, resulting in a list of 40 research priorities.

### 2.3 Round 3

The revised list of 40 research priorities (in English and French) was distributed to the working group, as well as to a broader group of 203 researchers and stakeholders who were registered to attend a meeting on “Treat All” organized by the IeDEA consortium in Kigali, Rwanda. This meeting involved researchers and clinic staff involved in IeDEA in East, Central, Southern and West Africa, along with representatives from the Government of Rwanda, HIV policy and implementation experts, donor representatives, other non‐IeDEA affiliated researchers, and representatives from four advocacy organizations representing the HIV community. Via a second Qualtrics survey, all 203 meeting registrants, including members of the working group, were invited to review and anonymously rate the list of 40 research priorities, using the same five‐point rating scale. Survey responses were received from 72/203 (36%) researchers from 14 countries. Mean ratings and the proportion of respondents rating each research priority a “5” (highest) in importance were calculated.

### 2.4 Round 4

At the Kigali meeting in November 2017, all 203 attendees (100%) actively participated in breakout sessions to further refine the research priorities. Meeting attendees self‐selected into three groups, each focused on a different 90‐90‐90 target (i.e. diagnosis, treatment, or viral suppression). Each group reviewed the list of 40 research priorities and their ratings from Round 3 and deliberated to reach consensus on five to seven priorities most critical for attaining the group's respective 90‐90‐90 target. Groups were also encouraged to modify the proposed priorities to reflect important topics that had not been identified *a priori*. This process resulted in a combined list of 16 research priorities.

### 2.5 Round 5

In follow‐up to the Kigali meeting, 21 members of the working group participated in two teleconference calls to review the research priorities recommended by the three breakout groups and to discuss areas of overlap and ways to combine research priorities. This step produced a final list of nine research priorities.

### 2.6 Round 6

The last round of the Delphi process was designed to assess the degree of consensus around the final list of research priorities. In January 2018, individualized survey links for a third and final online Qualtrics survey (in both English and French) were sent to all Kigali meeting participants and working group members. As with previous rounds, respondents were invited to rate each research priority on a five‐point scale in terms of its importance to Treat All implementation. Responses were received from 93/203 participants (46%), and mean ratings and the proportion rating each priority a “4” or “5” in importance were calculated.

### 2.7 Human subjects

The protocol for this project was reviewed by the Institutional Review Board (IRB) at the City University of New York School of Public Health.

## 3 Results

### 3.1 Rating of and consensus on research priorities

More than 200 individuals participated in one or more rounds of the research prioritization process, representing 12 countries in SSA (Burundi, Cameroon, Côte d'Ivoire, Democratic Republic of Congo, Kenya, Nigeria, Republic of the Congo, Rwanda, Senegal, South Africa, Tanzania and Uganda), as well as Europe (France and Switzerland) and North America (United States). While 123/203 (61%) of these individuals participated in at least one of the research priority rating surveys, the final list of nine research priorities was rated by 93 participants (response rate of 46%). Almost two‐thirds (60%) of those who participated in the final rating of research priorities were based in SSA, 12% of respondents were based in Europe, and 28% were based in the United States. One‐fifth of respondents completed the survey in French. Respondents’ backgrounds and areas of work included clinical research and implementation science (71%), service provision (25%), programme management (16%), policy‐making or advocacy (6%), and other (10%), with about one‐fourth reporting multiple areas (e.g. research and service provision, programme management and research). Respondents reported a mean of 10 years of experience working in HIV/AIDS, across various disciplines, including adult HIV (54%), adolescents (35%), infants and children (29%), key populations (27%), maternal and child health/prevention of mother‐to‐child transmission (26%), metrics and monitoring (18%), mental health disorders (12%), models of care (10%), substance use disorders (8%) and policy modelling (6%).

There was a high level of consensus across the final list of 9 research priorities, with 73.9% to 91.3% of respondents providing ratings of a “4” or “5” in importance (Table 1). In analyses stratified by respondent characteristics (e.g. identification as a researcher vs. non‐researcher, engagement in local/national policy‐making, residence in SSA, survey language), there were few differences in ratings, with the exception of Research Priority 1 (*Generate accurate national and sub‐national estimates of the number and proportion of persons living with HIV who are undiagnosed, disaggregated by age, sex, and population group)* which was rated higher in importance by members of the working group than other respondents (4.7 vs. 4.3, *p *<* *0.05; 100% vs. 81% rating as a “4” or “5”).

**Table 1:** Treat All research priorities

Generating metrics, estimates, and evidence to guide Treat All policies, planning, monitoring and evaluation, and intervention development, with key metrics disaggregated by age, sex and population group	Mean rating % rating “4” or “5” in importance for Treat All
1. Generate accurate national and sub‐national estimates of the number and proportion of persons living with HIV who are undiagnosed	Mean: 4.4 Rating “5”: 57.6% Rating “4” or “5”: 85.9%
2. Characterize and understand critical facilitators of and barriers to timely diagnosis, care linkage, antiretroviral therapy (ART) initiation, sustained care engagement, and ART adherence, particularly for key populations and underserved groups, including infants, adolescents and men	Mean: 4.7 Rating “5”: 76.1% Rating “4” or “5”: 91.3%
3. Develop and validate country‐specific policy models to support decision‐making around Treat All implementation	Mean: 4.2 Rating “5”: 41.8% Rating “4” or “5”: 78.0%
4. Develop and apply metrics that reflect the timeliness with which short‐term and long‐term HIV care continuum outcomes are achieved	Mean: 4.1 Rating “5”: 44.1% Rating “4” or “5”: 77.4%
5. Estimate the incidence and prevalence of HIV drug resistance, as well as switching from second to third‐line regimens at national and subnational levels	Mean: 4.2 Rating “5”: 45.2% Rating “4” or “5”: 82.8%
**Intervention effectiveness trials and economic evaluations to improve the rollout of Treat All and its effect on the achievement of 90‐90‐90 goals**	
6. Identify service delivery models and strategies to optimize uptake of HIV testing, including repeat testing and linkage to care for key and underserved populations	Mean: 4.3 Rating “5”: 50.0% Rating “4” or “5”: 85.9%
7. Identify service delivery models and strategies to reduce the time from diagnosis to ART initiation for key and underserved populations	Mean: 4.3 Rating “5”: 50.6% Rating “4” or “5”: 82.4%
8. Identify service delivery models and strategies to improve early and sustained viral suppression, early identification of drug resistance, and timely regimen switching	Mean: 4.5 Rating “5”: 62.0% Rating “4” or “5”: 89.1%
9. Identify screening, diagnostic, and treatment interventions for mental health and substance use disorders that can be integrated into HIV care to improve timely diagnosis, ART initiation, retention in care and viral suppression	Mean: 4.1 Rating “5”: 39.1% Rating “4” or “5”: 73.9%

### 3.2 Research priorities

The final research priorities are presented in Table 1, with illustrative research questions related to each priority presented in Table 2. Of the final list of nine research priorities, five priorities are focused on generating critical metrics, estimates and evidence needed to inform policies, planning, monitoring and evaluation related to Treat All implementation. Four priorities relate to the need to conduct focused effectiveness trials and economic evaluations to improve the rollout of Treat All. The research priorities reflect consensus around the need to more fully characterize the barriers faced by key populations and underserved groups along each step of the HIV care continuum and to identify programmatic strategies and tailored models of care that meet the preferences and needs of these populations. Finally, the research priorities highlight the need for enhanced metrics and data related to the timeliness of achieving short‐ and long‐term outcomes along the HIV care continuum, with particular attention to drug resistance and regimen switching.

**Table 2:** Illustrative research questions and possible methods to address them

Research questions	Methods
Research Priority 1: Generate accurate national and sub‐national estimates of the number and proportion of persons living with HIV who are undiagnosed	
What is the prevalence of undiagnosed HIV, particularly for key and priority population groups (e.g. MSM, SW, PWID, infants, adolescent, pregnant women, men), and what is the size of key population groups (e.g. MSM, SW, PWID) at national and subnational levels? How does the prevalence of undiagnosed HIV vary by sub‐national geographic area?	• Routine monitoring data; serosurveys, biobehavioural surveys; modelling.
Research Priority 2: Characterize and understand critical facilitators of and barriers to timely diagnosis, care linkage, ART initiation, and sustained care engagement and ART adherence, particularly for key populations and underserved groups, including infants, adolescents, and men	
What factors (individual, cultural, and structural/systems) influence timely diagnosis of HIV (i.e. at higher CD4 counts) and timely linkage to HIV care? How does this vary by sociodemographics and for key and underserved populations (e.g. MSM, SW, PWID, infants, adolescents, men)?	• Mixed methods approaches with PLWH, providers, and policy makers; implementation science/intervention studies; studies exploring new settings for HIV testing.
Research Priority 3: Develop and validate country‐specific policy models to support decision‐making around Treat All implementation	
What are the country‐specific health and economic outcomes, including cost‐effectiveness and budget impact, associated with Treat All implementation? How should interventions that address local implementation challenges (e.g. advanced HIV at entry to care; loss to follow‐up; acquired and developed viral resistance) be efficiently prioritized? What strategies can best engage local decision makers in mathematical model development and translation of model findings into policy?	• Mathematical modelling; cost‐effectiveness and other economic studies; stakeholder meetings; key informant interviews.
Research Priority 4: Develop and apply metrics that reflect the timeliness with which short‐term and long‐term HIV care continuum outcomes are achieved (i.e. early diagnosis, rapid linkage to care following diagnosis, rapid ART initiation following linkage, viral suppression within 4 weeks of ART initiation, and rapid achievement of sustained viral suppression)	
What is the most appropriate care cascade metric for Treat All and what metrics should be used to monitor it? Is it possible to develop a metric of time from infection to ART initiation? What is the optimal timing of ART initiation after diagnosis confirmation (e.g. immediately after diagnosis, after initial adherence counselling, etc.) for maximizing retention in care, adherence, and clinical outcomes, and how does this vary by population subgroup and co‐morbidities (e.g.patients with TB co‐infection, substance use and mental health disorders)?	• RCT or cluster RCT in real world implementation setting (vs. research setting).
Research Priority 5: Estimate the incidence and prevalence of HIV drug resistance, as well as switch to second‐ and third‐line regimens at national and subnational levels	
What is the prevalence of acquired and developed HIV drug resistance, and how does this vary across national, subnational and patient populations? What is the rate of switching to second‐ and third‐line regimens, and how does this vary by setting and by patient characteristics	• Routine monitoring data; surveys; targeted studies at sentinel HIV care sites.
Research Priority 6: Identify service delivery models and strategies to optimize uptake of HIV testing, including repeat testing and linkage to care, for key and underserved populations	
What testing strategies and settings (e.g. self‐testing, home‐, and community‐testing, etc.) are effective in improving timely HIV diagnosis, for sociodemographic and other key subgroups (e.g. MSM, SW, PWID), and underserved populations (infants, adolescents, men, sexual partners of HIV‐infected individuals)? Which testing strategies are most preferred by client subgroups? Which can minimize stigma‐related barriers to HIV testing? What clinic and community‐based strategies are effective in improving linkage to‐ and retention in care and sustained viral load suppression?	• RCT/cluster RCT; hybrid trial design; mixed methods; discrete choice experiments.
Research Priority 7: Identify service delivery models and strategies to reduce the time from diagnosis to ART initiation for key and underserved populations	
What clinic and community‐based strategies are effective in linking patients to care, particularly for key and underserved populations (e.g. MSM, SW, PWID; men and adolescents)? What clinic and community‐based strategies are effective in ensuring timely initiation of ART, particularly for key and underserved populations? What strategies are effective in addressing stigma‐related barriers to HIV care? Which service models are most preferred by client subgroups and care providers? Are strategies, such as integrated care, task‐shifting, and community‐ and home‐based services an efficient use of scarce resources under Treat All?	• Mixed methods; RCT/cluster RCT; hybrid trial design, cost‐effectiveness and other economic studies; discrete choice experiments.
Research Priority 8: Identify service delivery models and strategies to improve early and sustained viral suppression, early identification of drug resistance, and timely regimen switching	
What strategies are effective in ensuring early and sustained viral suppression, particularly for key populations and priority subgroups (e.g. MSM, SW, PWID; men, adolescents and infants)? How can service integration strategies be used to support sustained viral suppression, particularly for key populations and priority subgroups? What strategies are most effective in ensuring early identification of drug resistance, and timely regimen switching?	• Mixed methods; RCT/cluster RCT; hybrid trial design; cost‐effectiveness and other economic studies.
Research Priority 9: Identify screening, diagnostic and treatment interventions for mental health and substance use disorders that can be integrated into HIV care to improve timely diagnosis, ART initiation, retention and viral suppression	
What is the feasibility and acceptability of integrating screening, diagnosis, and treatment (pharmacological and non‐pharmacological) of mental health and substance use disorders (MH/SUD) and into HIV care delivered by lay healthcare workers? What are effective strategies of integrating mental health and substance use disorders screening, diagnosis, and treatment into HIV care, particularly for improving timely diagnosis, ART initiation, retention and viral suppression. How can effective models for screening, diagnosis, and treatment of MH/SUD within HIV clinic settings be scaled‐up? What are the health outcomes, economic costs, and cost‐effectiveness of integrating MH/SUD screening/diagnosis and treatment within HIV clinic settings compared to current standard of care?	• Mixed methods; RCT/cluster RCT; hybrid trial design, cost‐effectiveness and other economic studies.

#### 3.2.1 Research Priority 1: Generate accurate national and sub‐national estimates of the number and proportion of persons living with HIV who are undiagnosed

- *Context*: To achieve the “first 90” target, timely estimates of the number and proportion of persons with undiagnosed HIV infection are critical for countries to ensure that HIV testing programmes are targeted appropriately and efficiently 20, 21. One recent study that analysed population‐based Demographic and Health Survey (DHS) data from 16 SSA countries estimated that only 54% of people living with HIV (range across countries 26% to 84%) were aware of their status, contributing to delays in care enrollment and ART initiation 22. Men, adolescents, those with lower education levels, and the poorest individuals are less likely to be aware of their status, resulting in late initiation of treatment, as well as lower and later attainment of viral suppression 22. Infants less than 18 months of age are also at risk of delays in care enrollment and treatment initiation because of challenges in early infant diagnosis testing, especially in resource‐limited settings 23, 24. Estimating the “first 90” for key populations (e.g. men who have sex with men [MSM], sex workers [SW], people who inject drugs [PWID], etc.) is difficult in contexts with unknown population size estimates 20, 21.
- *Research approaches*: Population‐based studies, such as demographic and health surveys that evaluate the implementation of testing services, frequent (annual) targeted HIV sero‐prevalence surveys in sub‐national geographic areas, and biobehavioural surveys 20, may be necessary for monitoring this population‐level metric. Surveys should report estimates disaggregated by sex and age, with finer age disaggregations (e.g. two‐year age ranges) used for children and adolescents 25. Studies leveraging health service utilization data, including antenatal care and other sentinel surveillance‐based methods, can also provide information about specific populations. Systematically characterizing individuals diagnosed and enrolling in HIV care with advanced disease can also provide insights about which populations are not being reached with existing testing and surveillance strategies.

#### 3.2.2 Research Priority 2: Characterize and understand critical facilitators of and barriers to timely diagnosis, care linkage, ART initiation, and sustained care engagement and ART adherence, particularly for key populations and underserved groups, including infants, adolescents and men

- *Context:* Global data suggest that the timeliness of ART initiation, as measured by the level of immunodeficiency at the start of ART, is highly suboptimal in relation to WHO guidelines, particularly in SSA, where a recent analysis of data from 767,000 patients in 21 countries showed that median CD4 counts at ART initiation remained below 300 cells/mm^3^ in 2015 26. Infants, adolescents, and men, in particular, are more likely to initiate treatment late and to not be retained in care 24, 25, 27, 28.
- *Research approaches*: Important factors for further study include quality of care; policy and administrative requirements; costs of services, including user fees; HIV‐related stigma; integrated screening and treatment of other health conditions (i.e. non‐communicable diseases); and community‐ and home‐based services (e.g. home‐based self‐testing, provision of multi‐month medication supplies, etc.). Mixed methods approaches should be used to better understand these barriers and their relative contribution to delays in diagnosis, linkage, ART initiation, and viral suppression, as well as to losses along the care continuum, particularly for key populations. Additionally, mixed methods research, such as discrete choice experiments 29, 30, should be used to identify preferences (i.e. facilitators) that could improve timely uptake of testing, HIV care, and sustained care engagement. The magnitude and type of HIV‐related stigma, and its impact must be measured along the care continuum.

#### 3.2.3 Research Priority 3: Develop and validate country‐specific policy models to support decision‐making around Treat All implementation

- *Context:* The use of mathematical modelling techniques, coupled with detailed, individual‐level observational data, can inform Treat All policy questions, including the efficiency, prioritization and affordability of HIV‐related interventions 31. A strong modelling literature confirms that earlier ART initiation reduces morbidity and mortality, is cost‐effective compared to deferred ART initiation 32, 33, 34, 35, 36, 37, 38, 39, and prevents new HIV infections 32, 40, which may reduce population‐level economic costs 31. However, additional work is needed to develop and validate mathematical models that better reflect local clinical context (e.g. epidemiologic and clinical care data, economic costs), integrate local health system constraints (e.g. workforce capacity, antiretroviral stockouts, etc.), and areas of potential health system improvement (e.g. integrated care). Addressing these gaps will facilitate policy‐relevant modelling projections that inform cost‐effectiveness of individual interventions, the affordability of these interventions, and allocative efficiency across interventions when resources are constrained 31, 41, 42.
- *Research approaches*: The development of country‐specific mathematical models can provide projections of health and economic outcomes related to Treat All, including budget impact, which is essential information for programme planning and decision‐making. A key component of this effort is incorporating realistic model assumptions and inputs that reflect local treatment and care patterns. These include real‐world challenges and health system constraints surrounding late diagnosis, linkage to care, ART initiation, *de novo* development of viral resistance, sustained viral suppression, and reaching key and underserved populations, all of which can inform contextually relevant analyses on the cost‐effectiveness, affordability and prioritization of alternative interventions to inform local Treat All implementation. Evaluating approaches for engaging decision‐makers in mathematical modelling studies can facilitate translation of study findings into policy and practice. Validation of modelling approaches and the use of observational data sources for deriving appropriate model parameter estimates remain important research areas.

#### 3.2.4 Research Priority 4: Develop and apply metrics that reflect the timeliness with which short‐term and long‐term HIV care continuum outcomes are achieved (i.e. early diagnosis, rapid linkage to care following diagnosis, rapid ART initiation following linkage, viral suppression within four weeks of ART initiation, and rapid achievement of sustained viral suppression)

- *Context:* A priority for Treat All approaches is minimizing the time between HIV infection and sustained viral suppression. Shortening this period maximizes individual clinical benefit and reduces the risk of onward transmission, ultimately reducing both new infections and HIV‐related morbidity and mortality. Although the 90‐90‐90 targets and HIV care continua delineate important milestones, current metrics do not reflect the timeliness with which these key outcomes are achieved 43. In addition, in some settings, routine HIV viral load monitoring is infrequent, and pre‐ART CD4 count monitoring is declining in frequency with Treat All implementation 44, 45, which limits opportunities to evaluate individual and public health impacts of HIV programming. Four key metrics for assessing the timeliness of continuum milestones include: (1) median CD4 count at diagnosis 46, 47, care enrollment 48, 49, and ART initiation 26, 45, 50, 51; (2) time between diagnosis, enrollment 52, 53, 54; (3) time between enrollment and ART initiation 49; and (4) time to first HIV viral suppression and sustained HIV viral suppression.
- *Research approaches*: Metrics for this research priority could be generated from routinely collected, patient‐level, programmatic data. Where CD4 count and viral load data are not readily available for a large enough proportion of clinics and patients, it may be possible to produce estimates from a systematic sample of sites. Additionally, if such data are non‐existent because of unavailability of testing or a lack of systematic monitoring, sentinel sites could be established to do systematic CD4 count monitoring immediately prior to ART initiation and viral load monitoring following ART initiation in accordance with national protocols. Such metrics should be disaggregated by age, sex and population group (i.e. key and underserved populations, as well as pregnant and breastfeeding women) 55. Implementers and researchers should also consider disaggregating cascades by period of diagnosis or enrollment, so that short‐term outcomes of newly diagnosed persons and new enrollees can be differentiated from patients already enrolled in HIV care.

#### 3.2.5 Research Priority 5: Estimate the incidence and prevalence of HIV drug resistance, as well as switch to second‐ and third‐line regimens at national and subnational levels

- *Context*: Emerging HIV drug resistance, including transmitted resistance to NNRTI‐based first‐line ART regimens, is a growing clinical and public health concern 56, 57. As a result, many countries are rolling out dolutegravir‐based ART as first‐line therapy 58. While implementation of Treat All dictates that healthy patients with good immunological status and no clinical signs of disease initiate treatment, these patients may have suboptimal treatment adherence and lower rates of retention, raising risks for development of drug resistance and subsequent transmission of resistant virus 59. A recent review 57 and several modelling studies have raised concerns about the potential for an increasing rate of drug resistance associated with Treat All strategies 56, 60, 61. Routine viral load monitoring and assessment of treatment adherence are therefore essential for detecting virologic failure early and for limiting the development of drug resistance 45. Equally important are data on the impact of patient “churn” (i.e. recurring patient disengagement and re‐engagement in care) on viral suppression, disease progression, the emergence of viral resistance, and the durability of ART – particularly for the first‐line regimens that form the backbone of care in SSA. Such data are limited, particularly in settings where routine pre‐ART CD4 count monitoring has been discontinued because it is not required for treatment initiation.
- *Research approaches:* Patient tracing studies should consider including an array of evaluations, including care status, viral load, CD4 count and genotyping, to estimate the true frequency of these outcomes and to assess the effect of disengagement from care on viral resistance. As loss to care and patient churn vary by patient characteristics, disaggregating results by sex, age and other key demographics (e.g. pregnancy status) will be important for understanding the dynamics of treatment failure and drug resistance. In addition, the impact of reduced treatment adherence or interruption of ART on the development of drug resistance should be assessed and compared among different treatment regimens. Investigations should also explore early signs of non‐adherence (e.g. dose timing measures and drug level measurements) among patients who are traced after being lost from original clinic of enrolment. Models designed to capture trends and drivers of drug resistance development are important for predicting outcomes and assessing the effectiveness of different treatment and monitoring strategies 57.

#### 3.2.6 Research Priority 6: Identify service delivery models and strategies to optimize uptake of HIV testing, including repeat testing and linkage to care, for key and underserved populations

- *Context:* Stigma and discrimination remain important barriers to HIV testing 62, 63, 64, and about half of all people living with HIV do not know their status 22. Accordingly, closing the testing gap via differentiated service models, tailored approaches for populations at risk, and stigma reduction strategies is central to Treat All implementation 65. For example, men are a particularly important group for tailored testing strategies, as they are less likely to be tested for HIV until they become ill 66, 67, 68, 69, 70. A number of models show promise for optimizing uptake of HIV testing and screening, including home‐ and community‐based testing, index partner testing, the integration of HIV testing into multi‐disease community‐level health campaigns, the use of lay cadres to expand testing and linkage to care, and self‐testing 71, 72, 73, 74, 75, 76, 77, 78.
- *Research approaches:* Cluster randomized controlled trials (RCTs), rigorous programme evaluations, mixed methods studies on optimal timing of and barriers to repeat testing, and discrete choice experiments on preferences related to testing location (home or community based) and modalities (e.g. integration of HIV testing into other health services) will aid in identifying effective strategies that improve early diagnosis and linkage to care, particularly for underserved groups.

#### 3.2.7 Research Priority 7: Identify service delivery models and strategies to reduce the time from diagnosis to ART initiation for key and underserved populations

- *Context:* With the rollout of Treat All, the public health approaches that have been effective in the rapid scale‐up of treatment to date may not be sufficient for reaching the 90‐90‐90 targets and achieving epidemic control 79, 80. For the general population and key population groups (e.g. MSM, SW, PWID, those with mental health or other substance use disorders, etc.), stigma remains a barrier to HIV care, contributing to delays in ART initiation 63, 81, 82. Men, particularly, are not adequately served by traditional approaches, as they remain less likely than women to start ART 27, 83, 84, 85, 86 and they have more advanced disease than women when they start ART 27, 87, 88. Wide‐scale implementation of Option B+ may further increase gender disparities in access to ART, as has been reported in Malawi 89 and Mozambique 90. Differentiated models of community‐ and facility‐based care hold promise for reducing the burden of clinic visits for both clients and providers, while supporting ART initiation, adherence and retention in care and improving health system cost efficiencies 79, 91, 92, 93.
- *Research approaches:* Rigorous programme monitoring and evaluation, cohort studies, step‐wedge trials, and other implementation science approaches can be used to generate evidence on the effects of differentiated care models and integrated service delivery on patient outcome measures across diverse epidemic contexts and populations. Mixed methods studies would be useful to identify specific populations who remain underserved by conventional service delivery models and the role of HIV‐related stigma in limiting access for these groups. Data are also needed on the cost and efficiency of various models of HIV care delivery for priority and key populations.

#### 3.2.8 Research Priority 8: Identify service delivery models and strategies to improve early and sustained viral suppression, early identification of drug resistance, and timely regimen switching

- *Context:* Differentiated care strategies are essential for meeting the needs of underserved groups and key populations who do not access routine services and/or require additional support to achieve optimal HIV outcomes 75, 91, 94, 95, 96, 97. It is estimated that about 15% of patients on first‐line ART do not achieve viral suppression within 12 months 98, 99, with children and adolescents more likely to have elevated viral loads 100, 101, 102. Moreover, even when patients fail to achieve viral suppression on first‐line ART, rates of regimen switching are lower than expected, and loss to care rates are high 103, 104. Monitoring viral load and resistance is critical for ascertaining patients’ status and the impact of treatment programmes.
- *Research approaches:* Additional research is needed on barriers to viral suppression and regimen switching in response to regimen failure/toxicity. Research is also needed to identify differentiated care models, enhanced adherence counselling, enhanced patient monitoring or continuous quality improvement techniques that can address the “failure cascade” 103 and improve retention and viral suppression rates, particularly for children, adolescents, and underserved populations. Mathematical modelling studies can demonstrate the importance and cost‐effectiveness of routine viral monitoring and resistance monitoring 57. In addition, the validation of algorithms for predicting treatment failure can inform selective testing strategies for settings with limited resources/capacity for routine viral load monitoring. Treat All implementers and researchers can benefit from effectiveness evaluations for current strategies to ensure timely viral load monitoring and switching to second‐line ART regimens for those with detectable viral load. Qualitative research to explore reasons why providers do not switch patients to second‐line regimens after first‐line therapy failure are also important.

#### 3.2.9 Research Priority 9: Identify screening, diagnostic, and treatment interventions for mental health and substance use disorders that can be integrated into HIV care to improve timely diagnosis, ART initiation, retention and viral suppression

- *Context*: Mental health disorders (e.g. depression, post‐traumatic stress disorder) are highly prevalent comorbidities in PLWH, globally, with rates that exceed those in the general population 105, 106, 107. Substance use disorders (alcohol, injection drugs and non‐injection drugs) among PLWH are also a growing concern in SSA 108, 109, 110, 111, 112, 113, 114. While mental health and substance use disorders are associated with suboptimal HIV treatment outcomes, including late ART initiation, poor ART adherence, lack of viral suppression and increased AIDS‐related mortality 115, 116, 117, 118, 119, 120, 121, 122, 123, 124, 125, the coverage of screening, diagnosis and treatment services for these disorders is extremely limited in SSA 117, 126, 127. Key constraints include workforce shortages, limited training on mental health and substance use disorders, the lack of validated and culturally appropriate screening and diagnostic tools, as well as the lack of proven treatment interventions that can be integrated into HIV care and delivered by non‐specialists in contexts facing mental health and substance use workforce challenges 128, 129, 130, 131.
- *Research approaches:* The magnitude of mental health and substance use disorders merits further study, along with the effects of integrated treatment for these disorders on HIV outcomes under Treat All 105, 108. In addition, implementation science approaches should be used to assess the delivery, efficiency, and effects of existing intervention models in SSA settings in order to identify scalable, cost‐effective mental health and substance use interventions to improve HIV outcomes. Such initiatives should prioritize task‐shifting modalities that can be integrated into HIV clinics.

## 4 Discussion

With engagement and input from a diverse group of over 200 experts and stakeholders, our process yielded a set of research priorities that an overwhelming majority of the group agreed were important for the successful implementation of Treat All policies in SSA. The priorities highlighted by this process are broadly aligned with those identified by funding agencies, such as the National Institutes of Health 132 and The Global Fund 133, as well as the recent Lancet Commission on strengthening the HIV response 134.

A persistent programmatic challenge reflected in these research priorities is early diagnosis and linkage to care. Recent data demonstrate that PLWH in the SSA region continue to initiate ART late 26, indicating that many PLWH live for years before achieving first viral suppression. Late ART initiation has persisted 26, 27 and is preventing more rapid declines in HIV mortality and incidence in the region. Importantly, men, those experiencing multiple dimensions of stigma, and other underserved populations are being left behind as HIV treatment expands in SSA 63, 66, 67, 68, 69, 70, 81, 135. New age and sex‐disaggregated metrics and targeted strategies for earlier diagnosis (i.e. at higher CD4 counts) and linkage to care are needed. Although more real‐world data on timely ART uptake under Treat All implementation are needed, early evidence on ‘Treat All’ in SSA and evidence from previous HIV treatment guideline expansions suggest that if people are eligible for treatment when they link to care, they will start ART rapidly with early retention in care and viral suppression following ART initiation 2, 49.

Once treatment is initiated, there is a need for better metrics and monitoring related to sustained viral suppression, treatment failure and regimen switching (i.e. second‐ and third‐line regimens). There is particular concern under Treat All implementation that persons who are not experiencing any clinical signs or symptoms may be at higher risk of disengagement from care and poor treatment outcomes. Information on these outcomes – disaggregated by age, sex and disease stage – should be used to guide the development and deployment of differentiated care strategies to maximize sustained viral suppression and minimize the development of viral resistance.

Another major challenge is achieving optimal outcomes for key and underserved populations and those with mental health 105 and substance use disorders 108, making this a critical area for investigation. Country‐specific models and modelling studies can help support these efforts by characterizing the potential public health benefits to be gained through optimal implementation of Treat All 31.

In pursuing these research priorities, it is critical to utilize rigorous study designs (e.g. comparison groups whenever feasible/possible) and to specify implementation approaches, intervention components, and programme outcomes in order to support the replication and adoption of effective strategies. Ministries of health and donors can leverage programmatic implementation opportunities that can support advancement of the implementation science agenda around these priorities. Through early and effective engagement of decision‐makers, researchers and implementers can ensure that their findings are relevant and will be translated into policy, programmes and services that ensure that the individual and population health benefits of Treat All are realized sooner rather than later.

## 5 Strengths and limitations

The use of a Delphi approach in formulating and refining a list of research priorities to inform Treat All implementation leveraged the expertise of more than 200 researchers and partners who work across no fewer than 23 SSA countries, as well as the multi‐disciplinary perspectives of an extended network of implementation experts, researchers, policy‐/decision‐makers, advocates and other stakeholders. The process also facilitated the participation of researchers from both English‐ and French‐speaking contexts, with 20% of those participating in the final round being French‐speakers. Despite the diverse backgrounds of participants, there was a high degree of consensus in ratings of the research priorities across groups.

The Delphi approach provides a means of engaging diverse participants in a research prioritization process, however, sustaining participation across rounds is a known challenge 136, 137. In this initiative, participation varied considerably across rounds, with more than 200 participants involved in breakout sessions at the November 2017 meeting in Kigali to identify five to seven priorities most critical for each of the 90‐90‐90 targets from a list of 40 proposed priorities. While participation in online surveys in other rounds was lower, the overall number of participants increased with each survey round.

The Delphi method also allows for independent and decentralized input from a diverse group of participants 14. Nonetheless, the outcomes of the process are strongly shaped by those who are most engaged. In this undertaking, the initial working group was predominantly composed of IeDEA researchers with backgrounds in clinical and epidemiological research, rather than social science. For example, IeDEA research primarily focuses on outcomes of patients already diagnosed and enrolled in HIV care. While research related to HIV testing strategies, community care, technological innovation, and safer and more effective drugs are recognized as vitally important, the backgrounds of participants in this process resulted in a more emphasis on questions related to the second and third 90‐90‐90 targets. Thus, there may be important research priorities for some settings that are not reflected here.

## 6 Conclusions

While priorities for specific countries and contexts inevitably will differ, the priorities generated through this modified Delphi process reflect the consensus of a broad group of individuals actively engaged in addressing HIV throughout SSA. As Treat All gains momentum in the region, these research priorities highlight critical areas of inquiry with potential relevance for ministries of health, funders, normative bodies, and other research networks as they develop research agendas, programme strategies, and funding priorities to accelerate progress in meeting the 90‐90‐90 goals.

## Competing interest

Diane Addison, Rose S. Bono, Ellen Brazier, Stephany N. Duda, April D. Kimmel, Pamela N. Murnane, Denis Nash, Kara Wools‐Kaloustian, and Marcel Yotebieng report grants/funding from the U.S. National Institutes of Health (NIH) during the conduct of this work, including the NIH IeDEA funding; outside of this work, Kara Wools‐Kaloustian also reports grants from the Centers for Disease Control (CDC), and the CDC Foundation. There are no potential conflicts of interests for any of the manuscript authors.

## Authors’ contributions

Marcel Yotebieng and Denis Nash conceived of the project, and Ellen Brazier coordinated all rounds of the Delphi process. Diane Addison, Ellen Brazier, Barbara Castelnuovo, Craig R. Cohen, Morna Cornell, Stephany N. Duda, Olivia Keiser, April D. Kimmel, Kathryn E. Lancaster, Valeriane Leroy, Pamela M. Murnane, Denis Nash, Amobi Onovo, Angela M. Parcesepe, Jean d'Amour Sinayobye, Annette H. Sohn, Per M. von Groote, Rachel C. Vreeman, Gilles Wandeler, Radhika Wikramanayake, Carolyn F. Williams, Kara Wools‐Kaloustian, Constantin Yiannoutsos, and Marcel Yotebieng participated in calls and meetings of the Treat All Consensus Statement Working Group to define and direct the consensus development process. Robert Agler, Charlotte Bernard, Rose S. Bono, Ellen Brazier, Barbara Castelnuovo, Craig R. Cohen, Morna Cornell, Mary‐Ann Davies, Stephany N. Duda, Olivia Keiser, April D. Kimmel, Kathryn E. Lancaster, Pamela M. Murnane, Denis Nash, Amobi Onovo, Angela M. Parcesepe, Jennifer L. Syvertsen, Rachel C. Vreeman, Per M. von Groote, Radhika Wikramanayake, Kara Wools‐Kaloustian, Constantin Yiannoutsos, and Marcel Yotebieng conducted literature reviews, proposed initial lists of research priorities, and wrote/co‐wrote sections of the background document for the 2017 meeting in Kigali, Rwanda on Treat All implementation in sub‐Saharan Africa. Denis Nash, Ellen Brazier, and Marcel Yotebieng drafted the consensus statement manuscript, drawing on the background document prepared for the Kigali meeting. Diane Addison, Ellen Brazier, Barbara Castelnuovo, Craig R. Cohen, Morna Cornell, Stephany N. Duda, Olivia Keiser, April D. Kimmel, Kathryn E. Lancaster, Valeriane Leroy, Pamela M. Murnane, Denis Nash, Amobi Onovo, Annette H. Sohn, Rachel C. Vreeman, Per M. von Groote, Gilles Wandeler, Carolyn F. Williams, Kara Wools‐Kaloustian, and Marcel Yotebieng critically reviewed drafts and contributed substantive content to the consensus statement manuscript.

## Appendix 1

### Treat all Consensus Statement Working Group

Diane Addison, Keri Althoff, Ellen Brazier, Barbara Castelnuovo, Craig R. Cohen, Morna Cornell, Mary‐Ann Davies, Geraldina Dominguez, Stephany N. Duda, Aimee Freeman, Antoine Jaquet, Olivia Keiser, April D. Kimmel, Kathryn E. Lancaster, Valeriane Leroy, Janne Markus, Rosemary McKaig, Pamela M. Murnane, Denis Nash (co‐Chair), Dominique Nsonde, Amobi Onovo, Angela M. Parcesepe, Jean d'Amour Sinayobye, Annette H. Sohn, Per M. von Groote, Rachel C. Vreeman, Gilles Wandeler, Radhika Wikramanayake, Carolyn F. Williams, Kara Wools‐Kaloustian, Constantin Yiannoutsos, Marcel Yotebieng (co‐Chair).
